# Investigating the Design of ssPalmO-Derived Lipid Nanoparticles for mRNA Delivery Applications Using Molecular Dynamics Simulations

**DOI:** 10.1021/acsomega.5c05079

**Published:** 2025-12-10

**Authors:** Anjana Barange, Meitram Niraj Luwang, Santosh Kumar Meena

**Affiliations:** † Chemical Engineering and Process Development Division, 29616 CSIR-National Chemical Laboratory (NCL), Dr. HomiBhabha Road, Pune 411008, India; ‡ Department of Chemical Engineering, Indian Institute of Technology Ropar, Rupnagar, Punjab 140001, India; § Academy of Scientific and Innovative Research (AcSIR), Ghaziabad 201002, India

## Abstract

The rational design of lipid nanoparticles (LNPs) is essential for the effective transport of drugs and genetic material, as their structural and dynamic properties are heavily influenced by lipid composition and functional group modifications. In this study, we employed molecular dynamics simulations with density functional theory (DFT) derived force fields to investigate the bilayer properties of ssPalmO lipids, their phenyl ester (ssPalmO-phe) and benzyl ester (ssPalmO-ben) derivatives, as well as their *cis* and *trans* isomers. While all systems formed stable bilayers, *cis*-ssPalmO deviated by adopting a flexible, nonlamellar architecture. *Trans* isomers of ssPalmO-phe and ssPalmO-ben exhibited greater bilayer thickness, packing density, and order parameters due to stronger intramolecular chain interactions, while their aromatic substituents reduced lateral diffusion relative to ssPalmO. *Trans* isomers exhibited lower electrostatic potential differences, which increased upon incorporation of helper lipids, concomitantly enhancing bilayer packing and thickness while suppressing diffusion. These results clarify how lipid functionalization, stereochemistry, and helper lipid composition modulate bilayer organization, offering molecular level guidance for rational LNP design in drug and mRNA delivery.

## Graphical abstract

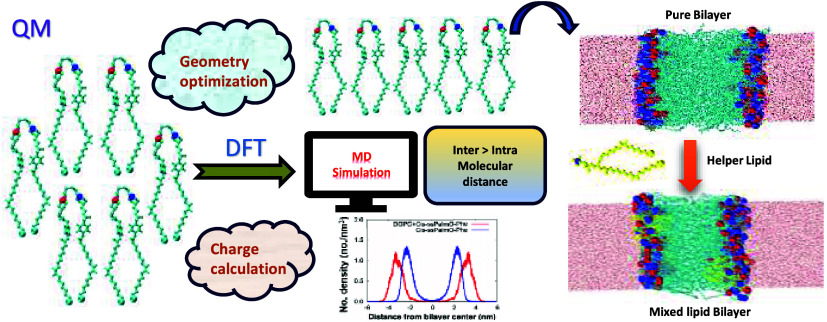

## Introduction

Viral vectors, such as adeno-associated virus (AAV), have been widely used for gene delivery; however, difficulties in large scale production, stability, and storage constrain their clinical application. These limitations have motivated the development of nonviral carriers, with lipid nanoparticles (LNPs) emerging as the most promising alternative owing to their biocompatibility, tunable size, and ability to encapsulate nucleic acids and small molecules.^1,2^ The concept of LNPs originated from liposome research in the 1960s, although early formulations displayed low encapsulation efficiency due to neutral lipids and passive loading methods.^3,4^ The use of cationic and subsequently ionizable lipids with p*K*_a_ values below 7 resulted in effective nucleic acid encapsulation and pH triggered endosomal release.^5,6^ In 1995, Doxil was approved for cancer therapy,^7^ and in 2018, ONPATTRO (patisiran) was approved as the first siRNA-LNP therapeutic.^8^ The success of mRNA-based LNP vaccines against SARS-CoV-2 further demonstrated the clinical potential of this platform.^9^

Lipid nanoparticles (LNPs) made of ionizable lipids, phospholipids, cholesterol, and PEG-lipids play a crucial function in RNA therapies, with helper lipids influencing their form, stability, and delivery effectiveness.^10^ Small structural or compositional modifications can significantly impact encapsulation, release, and activity,^11,12^ but experimental characterization is limited due to their complexity.^13^ Although LNPs frequently employ monolayer designs, bilayer models remain computationally tractable and experimentally validated for studying lipid–lipid interactions, chain conformations, and electrostatics. Recent advances in MD simulations, including Martini 3 coarse-graining,^14^ multiscale assembly studies,^15,16^ and atomistic investigations of clinically relevant lipids such as SM-102,^17^ provide molecular level insights that bridge experiments and theory. Combining these methods with DFT derived force fields enables the predictive design of ssPalmO-based systems and helps define the mechanistic principles for next-generation LNPs.

Molecular dynamics (MD) simulations are essential tools for dissecting lipid nanoparticle (LNP) behavior at molecular resolution, particularly given the computational limitations of simulating full, solvent explicit LNPs. While coarse grained (CG) models offer efficiency, they face challenges in accurately representing interactions among ions, lipids, solvents, and helper lipids.^18^ Bilayer models, though simplified, have proven invaluable: for example, lipid17 parameters clarified how ionizable lipids like MC3 influence bilayer structure,^19^ and CHARMM-GUI enhancements revealed how PEGylated lipids stabilize bilayers.^20^ Bilayer level insights on PEGylation, nucleic acid interactions, and pH sensitive phase behavior have deepened our understanding of drug loading and RNA release mechanisms.^21–25^ Although LNPs are often described as containing a single phospholipid monolayer surrounding a cargo core, mounting evidence shows that their internal architecture is far more heterogeneous, with lamellar, nonlamellar, and bilayer like domains emerging depending on lipid composition and pH. Consequently, bilayer models remain a widely accepted and computationally tractable surrogate to probe lipid–lipid interactions, packing, fluidity, and chain order, which critically influence encapsulation, stability, endosomal escape, and RNA release. Although bilayer systems of conventional ionizable and PEGylated lipids have been extensively studied, molecular level simulations of ssPalmO derivatives are lacking. These proton activated, redox cleavable lipids with their phenyl/benzyl substitutions, *cis*/*trans* isomerism, and helper lipid incorporation are expected to significantly influence bilayer properties and, consequently, nanoparticle performance. To address this gap, we performed atomistic MD simulations with DFT-derived force fields on bilayers of ssPalmO, ssPalmO-phe, ssPalmO-ben, and their *cis*/*trans* isomers, both alone and in helper lipid mixtures. By systematically quantifying bilayer thickness, packing density, diffusion, order parameters, and pH response, our work establishes direct structure–function correlations that provide mechanistic design principles for next generation ssPalmO based LNPs.

A particularly important class of ionizable and stimuli-responsive lipids is the disulfide cleavable, proton activated lipid like carrier (ssPalm) family. For example, ssPalm was developed to co-deliver sgRNA and Cas9 mRNA, enabling controlled RNA release in the reductive cytoplasmic environment.^26,27^ In these carriers, protonation of tertiary amines under acidic endosomal conditions induces a positive surface charge that promotes endosomal membrane interaction, while cytoplasmic disulfide cleavage facilitates cargo release.^28^ Structural modifications of ssPalm, such as adding myristic acid (ssPalmM) or vitamin scaffolds (ssPalmV, ssPalmA), have been employed to target tissues such as liver and renal cancer.^29–31^ More recently, oleic acid based ssPalm variants (ssPalmO) displayed anti-inflammatory characteristics and enhanced nucleic acid delivery efficiency. Further advancements were accomplished by inserting degradable linkers such as phenylalanine (ssPalmO-phe).^32,33^ Advanced versions, such as *cis*-ssPalmO-phe, have demonstrated improved transfection activity by destabilizing plasma and endosomal membranes, especially when paired with helper lipids.^34^

Designing lipid nanoparticles (LNPs) for therapeutic applications remains highly challenging due to the interplay of multiple factors, including lipid chemistry, phase behavior, toxicity, circulation time, phagocytic uptake, and nucleic acid release efficiency.^35,36^ Experimental optimization alone is resource intensive and often limited by the lack of molecular level insight into lipid organization, dynamics, and nucleic acid interactions. Molecular dynamics (MD) simulations have therefore emerged as a powerful tool to bridge this gap, providing a mechanistic understanding of lipid structural and dynamical behavior and guiding the rational design of improved LNPs.^37,38^ However, detailed theoretical studies on ssPalmO-based lipid bilayers are limited, particularly regarding the effects of functional modifications such as phenyl (ssPalmO-phe) and benzyl (ssPalmO-ben) substitutions, or *cis* and *trans* isomerization, in combination with helper lipid incorporation on bilayer structure and nanoparticle performance. To address this, we employ atomistic MD simulations with DFT derived force fields to study bilayers formed by ssPalmO and its derivatives, both individually and in combination with helper lipids. Our aim is to establish molecular level design principles and demonstrate how bilayer properties such as lipid packing density, bilayer thickness, ordering, diffusion coefficient, and electrostatic potential across the bilayer can be leveraged for the rational optimization of LNP formulations to achieve enhanced delivery efficiency.

## Computational Details

### DFT Calculations and Force-Field Development

The geometry optimization of all lipids in this study was performed using density functional theory (DFT) with the B3LYP^39,40^ exchange-correlation functional and the 6–311G+(d,p)^41^ basis set implemented in the Gaussian 09 program package.^42^ These optimized geometries of molecules were then further used to obtain the force field (FF) required to run classical MD simulations. The FF parameters for all the molecules were obtained by the procedure followed in ref^43^ However, the atomic partial charges used in the FF parameters of all the molecules were obtained after geometry optimization using the electrostatic potential (ESP) fitting method.^44^ The van der Waals parameters were taken from the GROMOS 53A6 parameter set.^45^ Similarly, FF parameters for DOPC was obtained by the procedure followed in ref 43, where the van der Waals parameters were taken from the GROMOS parameter set.^45^ The GROMOS force field is suitable for biomolecular systems, membrane formation, and transport over membranes.^45^ Although earlier versions of the GROMOS force field had certain limitations, the updated GROMOS 53A6 version introduced significant improvements through systematic reparameterization based on ab initio calculations and fitting to thermodynamic data. These refinements enhanced the agreement between simulations and experimental results for key structural properties such as area per lipid, electron density profiles, bilayer thickness, hydration, and acyl chain ordering and conformation across various lipid systems.^46^ The GROMOS force field was previously used for MD simulations of surfactants, ligands, and ions around gold nanorods and nano bipyramids.^47–58^ These simulations predicted micellar structures with varying surface densities on the tips and sides, depending on the exposed facets of the particles. In 2013, using MD simulations, we predicted that CTAB formed micelles separated by water–ion channels on different gold surfaces, providing a pathway for the diffusion of ions and Au reactants toward the goldsurfaces.^47^ These predictions were recently confirmed in 2024 using graphene liquid cell transmission electron microscopy,^59^ proving that the GROMOS force field gave an accurate description of the dynamics of the micelle even over the surfaces in solution. Experimental studies of various parameters of the lipid bilayers for the lipids reported in this study are currently unavailable. However, we compared the 1–2 bond pair distances of *Cis*-ssPalmO-ben obtained from our MD simulations with those derived from dissipative particle dynamics (DPD) simulations for the same lipid (see SI for details). The results show that our values are in close agreement with those from the DPD simulations.

### Models and Simulation Details

All the systems, pure and mixed, are obtained by packing the lipid molecule in a box using the packmol^60^ and solvating it with water molecules. Each lipid molecule modeled here has a net charge of zero, making the lipid system electrically neutral. Therefore, there is no need to add counterions to the simulation box. The details of each lipid bilayer, such as the number of lipids, the number of water molecules, and the box dimension, are given in Table 1.

**Table 1:** Details of the Composition of the Bilayer Model System for ssPalmO, ssPalmo-phe, and ssPalmO-ben and Their Isomers

sr., no.	name of lipids	no. of lipid molecules in an upper leaflet	no. of lipid molecules in the lower leaflet	total no. of lipids	no. of the water molecules	box dimensions (*x*,*y*,*z*) (nm)
1.	*cis*-ssPalmO-phe	75	75	150	17,662	6.1, 6.1, 24.1
2.	*trans*-ssPalmO-phe	75	75	150	19,154	6.2, 6.2, 25.6
3.	*cis*-ssPalmO-ben	75	75	150	15,597	5.4, 5.4, 25.8
4.	*trans*-ssPalmo-ben	75	75	150	18,348	5.5, 5.5, 27.5
5.	*trans*-ssPalmO	75	75	150	11,225	5.1, 5.1, 21.0
6.	*cis*-ssPalmO-phe+DOPC	69 + 6	69 + 6	150	19,186	6.2, 6.2, 22.0

All MD simulations were carried out using the GROMACS package (version 2018.1).^61^ After adding water, energy was minimized until the highest force applied to any one atom was less than 1000 kJ mol^–1^ nm^–1^. Within a cutoff length of 1 nm, the Lennard-Jones potentials for van der Waals interactions were computed. The electrostatic interactions were calculated using a summation algorithm, the particle-mesh Ewald (PME)^62^ up to a cutoff distance of 1.0 nm was employed for real space summation.^63^ Using the Parrinello–Rahman barostat, a semi-isotropic pressure coupling system was used to provide a pressure of 1 atm by a compressibility of 4.5 × 10^–5^ bar^–1^ as implemented in GROMACS during the simulations.^63^ A constant temperature of 303.15 K^34^ was maintained by using the Berendsen thermostat.^64^ Under periodic boundary conditions, the simulations were run in rectangular boxes using a Verlet cutoff scheme^65,66^ algorithm for integrating the Newtonian equation of motion at a time step of 2 fs, and trajectories were saved every 50 ps. We equilibrated the systems in four steps of NVT equilibration (constant particle number, volume, and temperature) for 1 ns each. Then the equilibrated NVT structure was subjected to NPT equilibration (constant particle number, pressure, and temperature), followed by four steps for equilibration, each for 1 ns. Final production run simulations were performed for 500 ns. Each system has two sets of final production simulations, each spanning 500 ns. The last 100 ns were used for analysis for each simulation.

## Results and Discussion

To understand the design of ssPalmO-derivative-based lipid nanoparticles for drug or mRNA delivery applications, we first investigated the structural and dynamic properties of lipid bilayers composed of various derivatives of the parent ssPalmO (Figure 1), specifically ssPalmO-phe, ssPalmO-ben, and their isomers, using molecular dynamics (MD) simulations. We compared key properties, including bilayer thickness, packing density, electrostatic potential difference across the bilayer, order parameters, and lateral diffusion coefficients.

**Figure 1:**
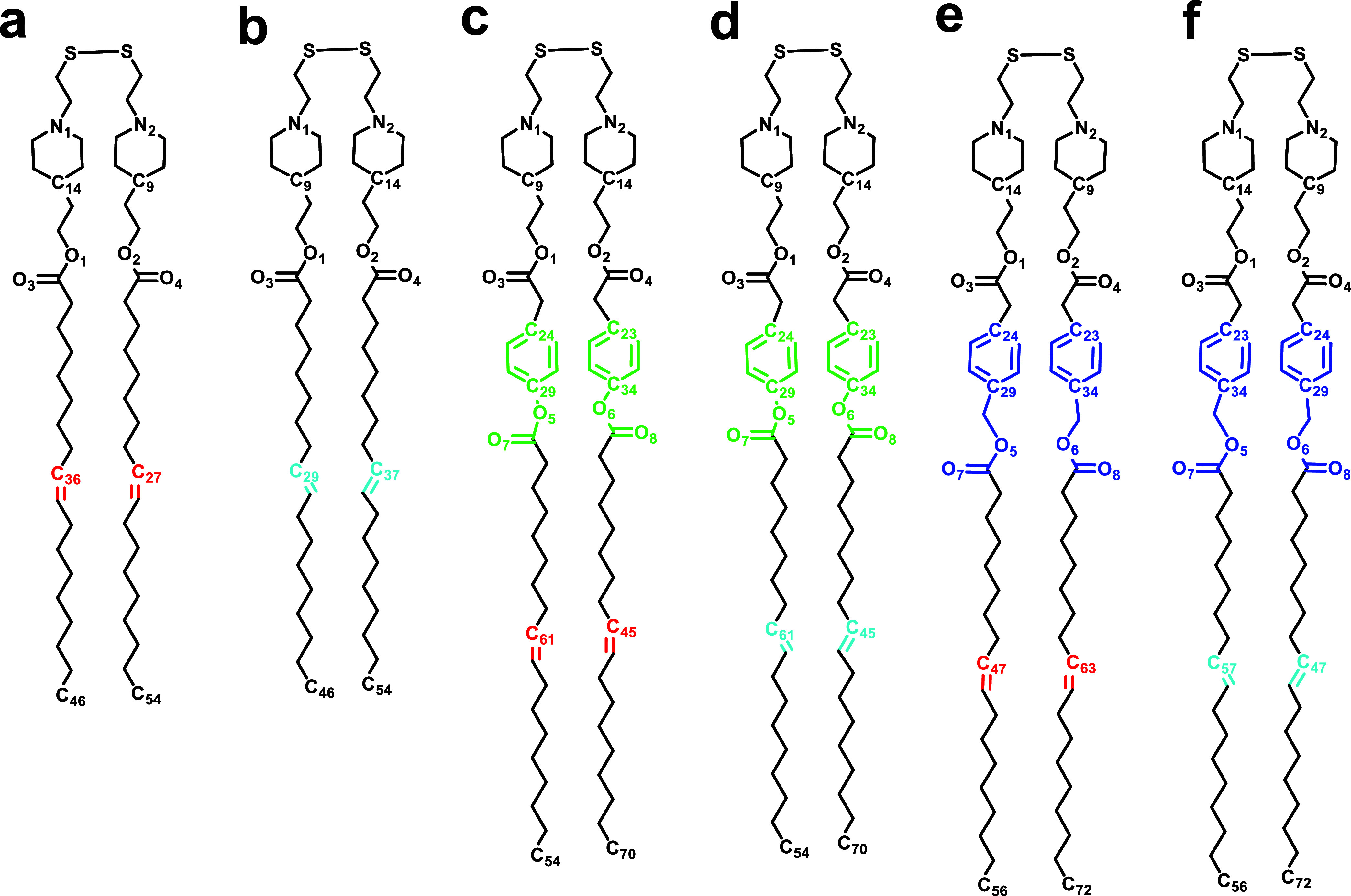
Chemical representation of the lipids and their isomers: (a) *Cis*-ssPalmO, (b) *Trans*-ssPalmO, (c) *Cis*-ssPalmO-phe, (d) *Trans*-ssPalmO-phe, (e) *Cis*-ssPalmO-ben, and (f) *Trans*-ssPalmO-ben.

For the design of mixed bilayers with the DOPC helper lipid, *cis*-ssPalmO-phe was selected, as *trans* fatty acid containing lipids are known to negatively impact membrane associated functions critical to human health, such as lipid metabolism, membrane protein activity, and cell signaling. The *cis* isomer of ssPalmO-phe is both self-degradable and biocompatible, while the *cis* form of ssPalmO-ben lacks self-degradability.^33^

The following sections present a detailed analysis of the structural and dynamic parameters of ssPalmO derivative based bilayers, as well as the behavior of mixed bilayers composed of *cis*-ssPalmO-phe and DOPC helper lipids, based on our simulation results.

### Bilayer Thickness

Snapshot from the simulation of the bilayer of *cis*-ssPalmO-phe, *trans*-ssPalmO-phe, *cis*-ssPalmO-ben, *trans*-ssPalmO-ben, and *trans*-ssPalmO in aqueous solution after 500 ns are shown in Figure 2. The number densities of lipid tails, head groups, and N atoms as a function of *Z*-axis for the bilayer of *cis*-ssPalmO-phe, *trans*-ssPalmO-phe, *cis*-ssPalmO-ben, *trans*-ssPalmO-ben, and *trans*-ssPalmO are also reported in Figure 2. In each bilayer, the number density of lipid tails has the highest peak at the center and drops to zero at the edges due to the arrangement of all lipid tails at the bilayer center. Similarly, the number density of head groups peaks at the edges, indicating that the head groups are positioned at the bilayer’s periphery.

**Figure 2:**
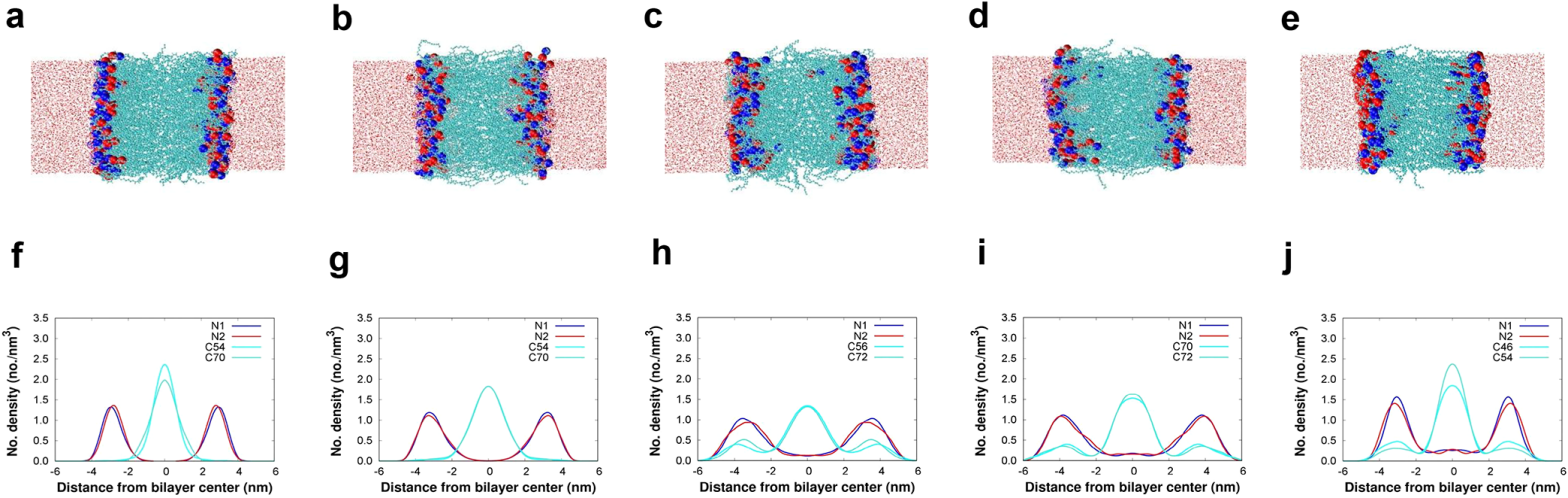
Snapshot from the simulation of the bilayer of (a) *Cis*-ssPalmO-phe, (b) *Trans*-ssPalmO-phe, (c) *Cis*-ssPalmO-ben, (d) *Trans*-ssPalmO-ben, (e) *Trans*-ssPalmO in aqueous solution after 500 ns. The number density of lipid tails (in cyan and turquoise), and head groups (in blue and red) as a function of distance from the bilayer center for (f) *Cis*-ssPalmO-phe, (g) *Trans*-ssPalmO-phe, (h) *Cis*-ssPalmO-ben, (i) *Trans*-ssPalmO-ben, (j) *Trans*-ssPalmO.

The snapshot from the simulation of the bilayer of mixed lipids (DOPC *+* *cis*-ssPalmO-phe) and number densities of lipid tails and head groups and N atoms as a function of *Z*-axis for the bilayer reported in Figure 3. The distance between the two peaks of the headgroup in number densities represents the bilayer thickness. The bilayer thickness for each lipid system is reported in Table 2. The thickness of the bilayer for *cis*-ssPalmO-phe, *trans*-ssPalmO-phe, *cis*-ssPalmO-ben, *trans*-ssPalmO-ben, *trans*-ssPalmO, and the mixed lipids (*cis*-ssPalmO-phe *+* DPOC) is 6.3, 6.4, 7.1, 7.2, 6.5, and 6.6 nm, respectively. Twice the equilibrium lengths of lipids in their straight form are also reported in the Table 2. The bilayer thickness of all lipids is approximately twice the equilibrium length of the lipid in its straight form.

**Figure 3:**
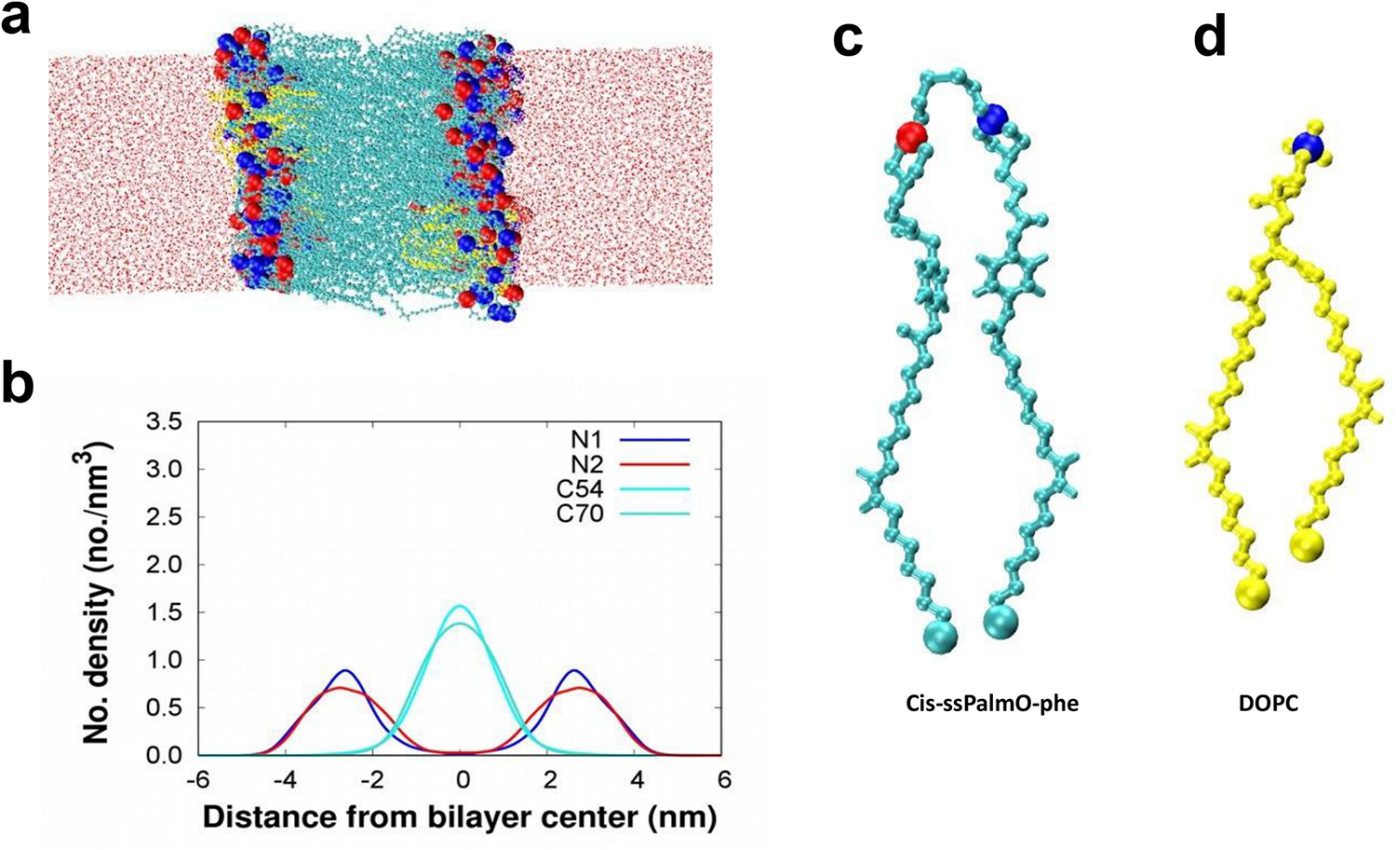
(a) Snapshot from the simulation of the mixed lipid bilayer of *Cis*-ssPalmO-phe and DOPC and after 500 ns (b) The number density of lipid tails (in cyan and turquoise), and head groups (in blue and red) as a function of distance from the bilayer center for the *Cis*-ssPalmO-phe (c) Structure of *Cis*-ssPalmO-phe, and (d) Structure of DOPC.

**Table 2:** Twice of Equilibrium Length of ssPalmO, ssPalmO-phe, ssPalmO-ben, and Their Isomers, Their Bilayer Thickness, Diffusion Coefficients, Packing Densities, and Electrostatic Potential Difference across the Bilayer

name	isomer	2 × equilibrium length of lipid [nm]	thickness of bilayer [nm]	diffusion coefficient 10^–7^ [cm^2^/s]	packing density [no./nm^2^]	electrostatic potential [V]
ss-PalmO-phe	*cis*	7.34	6.30 ± 0.05	0.0971 ± 0.01	1.98 ± 0.02	0.56 ± 0.04
ss-PalmO-phe	*trans*	7.44	6.40 ± 0.03	0.0817 ± 0.04	2.11 ± 0.02	0.36 ± 0.02
ss-PalmO-ben	*cis*	7.80	7.10 ± 0.06	0.0841 ± 0.01	2.58 ± 0.01	0.31 ± 0.03
ss-PalmO-ben	*trans*	7.88	7.20 ± 0.06	0.0824 ± 0.01	2.73 ± 0.01	0.29 ± 0.02
ss-PalmO	*trans*	6.20	6.5 ± 0.08	0.1655 ± 0.01	2.78 ± 0.07	0.57 ± 0.03
ssPalmO-phe + DOPC	*cis*	7.34	6.60 ± 0.04	0.0536 ± 0.01	2.01 ± 0.01	0.93 ± 0.05

Bilayer thickness plays a crucial role in determining the stability, drug release rate, biocompatibility, and interactions with biological membranes.^67–69^ Achieving an optimal bilayer thickness is essential for effective drug delivery, as it enhances membrane stability, prevents premature drug leakage, and ensures efficient delivery to the target site.^70,71^ A thinner bilayer can enhance biocompatibility by minimizing potential immune responses and may facilitate faster drug release at the target site. Bilayer thickness is a key factor in regulating the timing and efficiency of drug delivery.^72,73^ Due to differences in equilibrium lengths, the isomers of ssPalmO-phe and ssPalmO-ben exhibit variations in bilayer thickness, with the *trans* forms being slightly thicker than their corresponding *cis* forms. This trend is consistent with observations by Kulig et al., who reported similar behavior for POPC (*cis*) and PEPC (*trans*) in pure bilayers.^74^ While the bilayer thickness of ssPalmO-phe is initially lower in its pure form, it increases upon the addition of helper lipids, approaching approximately twice the molecular length. This increase is attributed to the stabilizing interactions between the ssPalmO-phe and the helper lipids, which help maintain the lipid molecules in a more extended, linear conformation.

### Diffusion Coefficient

The lateral diffusion coefficient of lipids within a bilayer provides insight into the bilayer’s viscosity and fluidity key properties that influence drug or gene delivery processes.^75–78^ This coefficient affects how drug or mRNA molecules enter, exit, and move within lipid nanoparticles (LNPs). Its value is determined by several factors, including the bilayer’s composition, thickness, packing density, and temperature.

The diffusion coefficient of ss-PalmO-phe (*cis* and *trans*) and ssPalmO-ben (*cis* and *trans*) is lower compared to the original *trans*-ssPalmO lipid (Table 2). The lower diffusion coefficient observed in the ssPalmO lipid derivatives may be attributed to the presence of aromatic rings within their structures. Specifically, the phenyl ester (ssPalmO-phe), which acts as a self-degradable component, and the benzyl ester (ssPalmO-ben), a nondegradable counterpart, contribute to restricted lipid mobility by increasing structural rigidity. Additionally, the diffusion coefficient of the mixed lipid (*cis*-ssPalmO-phe + DOPC) is decreased when the helper lipid, like DOPC, is added to the bilayer than that of *cis*-ssPalmO-phe, indicating that the diffusion coefficient of ssPalmO-phe decreases upon introducing the helper lipid.

The diffusion coefficient of lipids in a lipid bilayer plays a crucial role in mRNA encapsulation and controlled release in lipid nanoparticles. A lower diffusion coefficient enhances lipid nanoparticle stability and minimizes premature mRNA leakage but may hinder efficient encapsulation and slow controlled release. Conversely, a higher diffusion coefficient improves encapsulation efficiency and facilitates mRNA release but may lead to premature leakage and reduced nanoparticle stability. Therefore, an optimal diffusion coefficient is essential for maintaining a balance between the stability of lipid nanoparticles, effective encapsulation, and efficient release of drugs or mRNA.

### Packing Density

The packing density, defined as 1/(Area per lipid),^79^ is widely used to monitor phase transitions and describe how tightly the lipids are packed in the bilayers. The average area per lipid is defined as the cross-sectional area of the whole system along the bilayer surface plane (*xy*-plane) divided by the number of lipids in each leaflet. The area per lipid gives insight into the packing of the bilayer and verifies the state of system equilibrium. In each bilayer leaflet, approximately half of the lipids are present. Consequently, the bilayer area, determined by the box’s dimensions, and the total number of lipids over the total cross-sectional area of the bilayer represent the packing density.^80,81^

The *trans* forms of ssPalmO-phe and ssPalmO-ben lipids exhibit higher packing density compared to their respective *cis* forms (Table 2). The parent molecule ssPalmO in its *trans* form has a packing density value of 2.78 lipids/nm^2^, which is comparably higher than its derivative lipids, which have aromatic rings in their structure.

However, the packing density of parent *trans*-ssPalmO, *trans*-sspalmO-phe, *trans*-ssPalmO-ben, and mixed lipid is higher than that of pure DOPC, DOPE, POPC, and PEPC, which have the packing density values 1.45, 1.59, 1.47, and 1.51, lipids/nm^2^ respectively.^74,82^ The *trans* lipid has a higher packing density than its *cis* form because the distance between intramolecular chains is shorter in the *trans* form. The shorter intramolecular chain distance observed in the *trans* form compared to the *cis* form is mainly due to their structural differences. The *trans* form has a more extended and straight conformation, while the *cis* form is bent. Because of this, the atoms or segments within the *trans* chain are naturally closer together in space. Therefore, the shorter distance does not necessarily mean that the intramolecular interactions are stronger in the *trans* form, it may simply reflect the natural geometry of the chain (Table 3). The average intramolecular chain distances are reported in Table 3 (and represented in Supporting Information Figure S1). The average intermolecular chain distance was calculated from the radial distribution function (RDF) (in Supporting Information Figure S2) and reported in Table 4. The distance between intermolecular chains is almost similar in all the lipid bilayers (Table 4) and shorter than the distance between intramolecular chains in all the lipid bilayers (Table 3). This suggests that intermolecular chain interactions are stronger than intramolecular chain interactions in all lipid bilayers.

**Table 3:** Average Intramolecular Chain Distances in nm between the Atoms within the Molecule^a^

lipids	N1–N2	C9–C14	O1–O2	O3–O4	C23–C24	C29–C34	O5–O6	O7–O8	Unsat.C	terminal C
*cis*-ssPalmO-phe	0.66	0.93	1.16	1.28	1.27	1.29	1.32	1.35	1.43	1.61
*trans*-ssPalmO-phe	0.65	0.92	1.12	1.20	1.22	1.25	1.27	1.31	1.33	1.54
*cis*-ssPalmO-ben	0.68	1.04	1.43	1.58	1.71	1.86	1.89	2.12	2.41	2.65
*trans*-ssPalmO-ben	0.69	0.97	1.38	1.53	1.69	1.81	1.61	2.01	2.37	2.51
*trans*-ssPalmO	0.64	0.90	1.12	1.22	–	–	–	–	1.54	1.59
ssPalmO-phe + DOPC	0.66	0.91	1.14	1.21	1.26	1.27	1.28	1.32	1.41	1.61

a The maximum standard error is 0.001 nm.

**Table 4:** Average Intermolecular Chain Distances in nm Calculated from RDFs Reported in Supporting Information Figure S2 ^a^

lipids	N1–N2	unsaturated carbon	terminal carbon
*cis*-ssPalmO-phe	0.55	0.52	0.41
*trans*-ssPalmO-phe	0.52	0.51	0.40
*cis*-ssPalmO-ben	0.54	0.53	0.46
*trans*-ssPalmO-ben	0.51	0.52	0.44
*trans*-ssPalmO	0.54	0.51	0.41
ssPalmO-phe + DOPC	0.54	0.52	0.41

a The maximum standard error is 0.001 nm.

On adding helper lipids, the packing density of *cis*-ssPalmO-phe increases (Table 2). The packing density significantly impacts bilayer properties such as permeability, thickness, membrane fluidity, molecular interactions, phase behavior, and overall biological functionality.^71,83,84^ Notably, the permeability of the lipid bilayer to various substances including ions, small molecules, drugs, and mRNA is strongly influenced by how tightly the lipids are packed. Lipid bilayers with lower packing density tend to be more fluid, allowing lipid molecules to move more freely, which can enhance permeability and facilitate the transport of molecules across the membrane.^85,86^

Hydrophobic interactions in these lipids primarily arise from van der Waals forces, which enable lipid molecules within a bilayer to interact with one another.^87^ The nature and strength of these interactions can vary with changes in packing density, directly influencing the organization and stability of the membrane.^88^ Variations in packing density reflected in the area per lipid can also significantly affect the phase behavior of lipid bilayers. Depending on factors such as temperature and lipid composition, bilayers can exist in different phases, such as the gel phase or liquid crystalline phase.^89^ Alterations in packing density may trigger phase transitions, thereby modifying the membrane’s physical properties.

An optimal packing density is essential for the biological functionality of lipid bilayers. As key structural components of cell membranes, lipid bilayers are involved in critical processes such as cell signaling, molecular transport, and structural integrity. The packing density of lipids within the membrane significantly influences properties like receptor mobility, membrane fusion, and protein–lipid interactions factors that are vital for these biological functions. In the context of drug and gene delivery, packing density also plays a critical role in the encapsulation and release of genetic material, such as mRNA, rRNA, oligonucleotides, and therapeutic drugs. Lower packing density increases free volume within the bilayer, enhancing the stability of hydrophilic cargo and facilitating the inclusion of hydrophobic drugs. In contrast, higher packing density reduces available space within the bilayer or core, limiting the encapsulation capacity. However, achieving efficient encapsulation and delivery is not governed by packing density alone. Other lipid nanoparticle components-such as helper lipids, cholesterol, and PEGylated lipids also play essential roles. While higher packing density slows the release of genetic material or drugs by decreasing membrane permeability, lower packing density promotes faster release through diffusion.^90–92^

### Electrostatic Potential

The electrostatic potential across the bilayer is calculated using the procedure followed in our previous work,^47^ where the Poisson equation is used to determine the electrostatic potential, which is given by the following expression

1 $$\dot{V\left(z\right)=-\frac{1}{\varepsilon_{0}\;}\int_{0}^{z}\;dz^{\prime }\;\int_{0}^{z\prime }\;\rho \left(z^{\prime \prime }\;\right)dz^{\prime \prime }\;}$$

where, ρ is the charge density. The potential *V*(*z*) and the charge density ρ­(*z*) are functions of position along the *z*-axis, perpendicular to the gold surface and the surfactant layer. Equation 1 has already been averaged over *x* and *y* coordinates. The simulation box is divided into thin slices along the *z*-axis, and the charge density of each slice is determined as the sum of the partial charges of all atoms within the slice over the slice volume. The electrostatic potential is set to zero at the beginning of the box (*z* = 0).

The potential difference value across each lipid bilayer is reported in the Table 2. *Cis* and *trans*-ssPalmO-phe have 0.56 and 0.36 V electrostatic potential difference across the bilayer. The ssPalmO-ben in *cis* and *trans* forms have 0.31 and 0.29 V, respectively. The potential difference across the bilayer is higher for the *cis* form of ssPalmO-phe and ssPalmO-ben. The parent molecule in the *trans* form has a potential difference of about 0.57 V. The electrostatic potentials of all the lipid bilayers simulated in this study range from approximately 0.3 to 0.6 V. However, the incorporation of helper DOPC lipids in the *cis*-ssPalmO-phe bilayer increases the electrostatic potential difference across the membrane from 0.56 to 0.93 V. These results indicate that when other helper lipids and cholesterol molecules are added, then the potential difference value could change, and that will affect the insertion of the drug or genetic molecule in the bilayer. The electrostatic potential value affects other properties like electrostatic interaction between the drug and nucleic acid base material, ion permeability, and cellular uptake, affecting the interaction between drug molecules and cell membranes.

The distribution of charges within a lipid bilayer and the presence of charged molecules or ions in the surrounding environment are the sources of the electrostatic potential across a bilayer. Although the lipid bilayer is normally electrically neutral overall, the bilayer may have an uneven electrostatic potential due to the distribution of charges among the lipid headgroups and the presence of charged molecules nearby.

### Deuterium Order Parameter

The order parameter (*S*_CD_) was calculated to investigate the chain mobility, orientation, and orderliness of lipid tails in lipid membrane systems.^93^ The *S*_CD_ value of carbon atoms next to the lipid headgroup typically increases and decreases along the carbon chain length. This results from the restricted motions of the lipid headgroup while the bilayer’s tail sections move more freely and are less ordered. The order parameter is evaluated from the eq 2.

2 $$S_{\mathrm{CD}}=\frac{1}{2}\left\langle 3\mathrm{co}s^{2}\theta -1\right\rangle$$

where, θ is the angle between the *z*-axis (the membrane normal) of the simulation box and the vector Cn–1 to Cn+1, and the brackets denote an average over all lipids. The order parameters for various lipid bilayers as a function of the number of carbons are reported in Figure 4b,c for the SN1 and SN2 chains, respectively. Both SN1and SN2 chains are the same; hence, they show nearly the same result. In general, the *S*_CD_ value of carbon atoms next to the lipid headgroup is higher and decreases as the carbon chain lengthens. The reason for this is that the lipid headgroup limits the motion, whereas the bilayer’s tail portions move with relative freedom.^94^ The lipid having an aromatic ring inserted in its chemical structure has a lower order parameter value than its parent lipid ssPalmO. The parent ssPalmO molecule in *trans* form is in a higher order than both the ssPalmO-phe and ssPalmO-ben lipid molecules. The *cis* form of the ssPalmO-ben lipid is less ordered among all the lipids. The *trans* form of all lipids has a higher order parameter value than their *cis* form. On average, the deuterium order parameter shows a slight decrease after the addition of a helper lipid such as DOPC in the *cis* ssPalmO-phe lipid bilayer.

**Figure 4:**
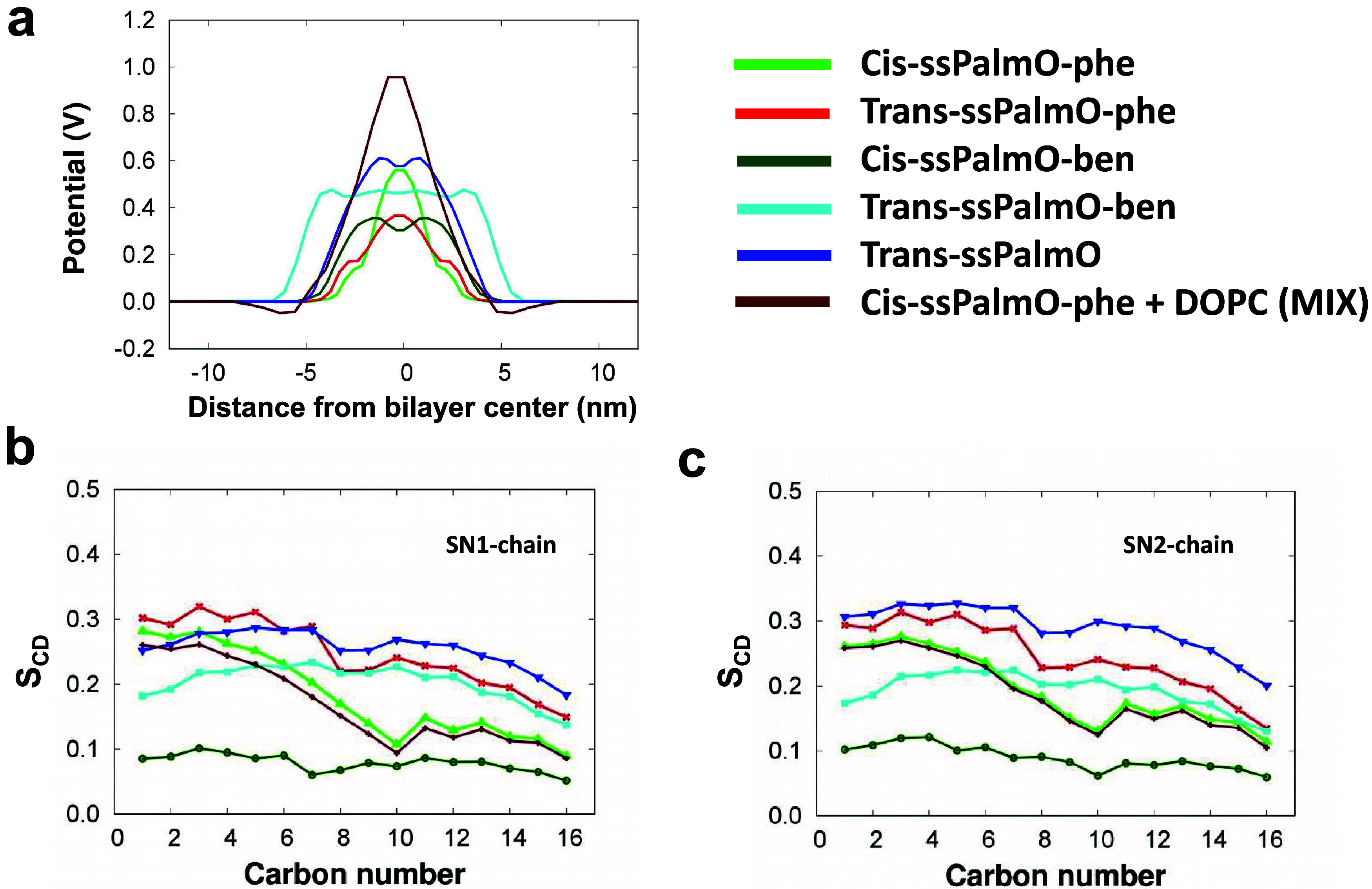
(a) Electrostatic potential for the simulated system, and deuterium order parameter for ssPalmO, ssPalmO-phe, and ssPalmO-ben and their isomer which are studied in this work (b) *S*_CD_ parameter for SN1 acyl chain of all simulated system, and (c) *S*_CD_ parameter for SN2 acyl chain of all simulated system.

Lipid acyl chain order parameters can be extracted from simulations and NMR experiments.^95^ Experimentally obtained order parameters for other lipids such as Dimyristoylphosphatidylcholine (DMPC)^96^ and 1-palmitoyl-2-oleoyl-*sn*-glycero-3-phosphocholine (POPC)^97^ have been reported in the literature. For DMPC, the order parameter values are lower near the headgroup, increase as one moves away from the headgroup, and then decrease again toward the tail. In the case of POPC, the order parameter of the Sn1 chain is higher near the headgroup and decreases gradually toward the tail, while the SN2 chain exhibits a distinct dip in the middle due to the presence of an unsaturated carbon. For ssPalmO derivatives and their isomers, our results suggest that the order parameter of carbon atoms adjacent to the headgroup is relatively high due to the disulfide bond linking both leaflets at the headgroup region, and it decreases progressively along the carbon chain. This trend arises because the headgroup region restricts motion, while the tail region experiences greater flexibility. A slight dip in the order parameter is also observed, similar to the SN2 chain of POPC, due to the presence of an unsaturated bond.

Changes in membrane fluidity are not always reflected directly by increases or decreases in these parameters. Some molecules, such as certain drugs or genetic materials, may increase order yet still promote faster lipid diffusion.^98^

Nucleic acid–based therapeutics have emerged as promising tools, with mRNA vaccines against COVID-19 demonstrating transformative potential in the treatment of a variety of disorders. However, due to their instability and polyanionic nature, nucleic acids require specialized delivery mechanisms, lipid nanoparticles (LNPs) currently representing the most effective strategy. Despite significant progress, the molecular-level understanding of LNP structure and function remains limited, and only a small portion of cargo is efficiently delivered, highlighting the need for rational, simulation-guided design of next-generation formulations.

In this study, we used molecular dynamics simulations to guide the rational design of lipid nanoparticles. We analyzed key properties, including number density, bilayer thickness, diffusion coefficient, packing density, electrostatic potential, order parameters, and intra- and intermolecular interactions. These were evaluated for bilayers made of pure ssPalmO derivatives and mixtures of *cis*-ssPalmO-phe with DOPC.

Our results show that intermolecular chain distances (Table 4) are consistently shorter than intramolecular (Table 3) ones across all lipid bilayers, indicating stronger intermolecular interactions throughout.

Additionally, the *trans* forms of ssPalmO-phe and ssPalmO-ben show greater bilayer thickness and packing density compared to their *cis* counterparts. This is likely due to shorter intramolecular chain distances in the *trans* forms, suggesting stronger intramolecular interactions. Overall, bilayer thickness, packing density, and lipid tail order parameters appear to be closely linked to intramolecular interactions.

The lateral diffusion coefficients of the *cis* and *trans* forms of ssPalmO-phe and ssPalmO-ben are comparable. However, these values are lower than those of ssPalmO due to the presence of the phenyl ester (phe) and benzyl ester (ben) ring groups in ssPalmO-phe and ssPalmO-ben. Furthermore, the electrostatic potential difference across the lipid bilayer is lower in the *trans* forms of ssPalmO-phe and ssPalmO-ben compared to their *cis* forms.

Upon the addition of the helper lipid DOPC, the bilayer thickness, packing density, and electrostatic potential of *cis*-ssPalmO-phe increased. This is primarily because the intramolecular chain distance in *cis*-ssPalmO-ben within the mixed bilayer is smaller than in other lipids and its pure form (Table 3).

Further, DFT calculations were conducted to explore the electronic properties of ssPalmO-phe in both *cis* and *trans* conformations.^99^ As shown in Figure S4 and Table S2, the *trans* isomer displays a smaller band gap of 4.74 eV and lower chemical hardness of 2.37 eV in comparison to the *cis* isomer, which has values of 4.91 and 2.46 eV, respectively. These findings indicate a greater softness and polarizability in the *trans* form, highlighting an enhanced intramolecular electron delocalization. This observation aligns with MD simulations, which shows that the *trans* conformation exhibits increased bilayer thickness, higher packing density, shorter chain lengths, and improved lipid tail ordering.

Also, the 2H NMR spectroscopy can be employed to investigate order parameters, while small-angle X-ray or neutron scattering (SAXS/SANS) and reflectometry techniques can directly evaluate bilayer thickness and packing density. The lipid diffusion behavior predicted by MD simulations can be validated against experimental data obtained from fluorescence recovery after photobleaching (FRAP) or single particle tracking. Collectively, these techniques provide a robust framework for experimentally validating the structural and dynamic features of bilayers predicted by our computational models.

Additionally, the ADMET study summarized in Table S3, which shows the ssPalmO derived lipids are extremely efficient carriers of lipid nanoparticles (LNPs). Their limited tissue diffusion, low clearance, and strong plasma protein binding promote long-term systemic stability and precise delivery of small medications or nucleic acids. Their moderate metabolic stability and low genotoxicity and carcinogenicity risks contribute to their excipient safety and biocompatibility. All of these characteristics work together to make ssPalmO based lipids ideal structural elements for LNP mediated therapeutic delivery as opposed to pharmacologically active compounds.

## Conclusions

In conclusion, MD simulations were performed on the pure forms of various derivative lipids of the parent ssPalmO and their isomers to explore the rational design of lipid nanoparticles as carriers for drugs or genetic material. All lipids, ssPalmO, ssPalmO-phe, ssPalmO-ben, and their isomers formed stable bilayers, except for the *cis* form of the parent ssPalmO lipid, which displayed high flexibility and adopted a nonlamellar structure. The bilayer thickness and packing density of the *trans* forms of ssPalmO-phe and ssPalmO-ben are higher than those of their *cis* counterparts due to variations in chain conformation and stronger intramolecular chain interactions in the *trans* bilayers. Our results suggest that bilayer thickness, packing density, and the deuterium order parameter of lipid tails are directly correlated with intramolecular lipid chain interactions.

The lateral diffusion coefficients of the *cis* and *trans* forms of ssPalmO-phe and ssPalmO-ben are lower than those of ssPalmO, likely due to the presence of the phenyl ester (phe) and benzyl ester (ben) ring groups in ssPalmO-phe and ssPalmO-ben. Additionally, the electrostatic potential difference across the lipid bilayer is lower in the *trans* forms of ssPalmO-phe and ssPalmO-ben compared to their *cis* forms.

Furthermore, our simulation results indicate that introducing helper lipids into the *cis*-ssPalmO-phe bilayer increases the packing density, bilayer thickness, and electrostatic potential difference across the bilayer while decreasing the lateral diffusion coefficient. These findings suggest that both the incorporation of functional groups into the main lipids and the composition of main lipids and helper lipids influence key bilayer properties, such as bilayer thickness, packing density, diffusion coefficient, and order parameters. Consequently, these changes may impact the stability, encapsulation efficiency, and release profile of lipid nanoparticles for drug or mRNA delivery. Our results provide valuable insights into the role of different lipids, helper lipids, and lipid functional groups in the rational design of lipid nanoparticles for the delivery of therapeutic agents and genetic material.

Such findings can guide the selection and optimization of lipid components for designing lipid nanoparticles to improve mRNA encapsulation efficiency, particle stability, membrane flexibility, and controlled release. For example, tuning packing density and order parameters can modulate membrane rigidity and fluidity, while insights into electrostatic potential and diffusion profiles can enhance mRNA lipid interactions, cellular uptake, and endosomal escape. Overall, this study provides a predictive framework for the rational design of next generation lipid nanoparticle formulations with improved delivery efficiency and therapeutic performance, thereby enabling hypothesis driven formulation strategies and reducing reliance on empirical, trial and error approaches in mRNA therapeutic development.

## Supplementary Material



## Notes

Details for the Intramolecular, Intermolecular distances calculations of atomic pairs, simulation details of single lipid, comparison of 1–2 bond pair distances of *cis*-ssPalmO-ben obtained from our MD simulations with those derived from dissipative particle dynamics (DPD), DFT calculation of ssPalmO-phe lipids, chemical structure of lipids and ADMET prediction (PDF)
