# Supplementary material for: Investigating the Design of ssPalmO-Derived Lipid Nanoparticles for mRNA Delivery Applications Using Molecular Dynamics Simulations
Source: ACS Omega. 2025 Dec 10;10(50):61256–67. doi: 10.1021/acsomega.5c05079 (PMC12750239; doi:10.1021/acsomega.5c05079)
Supplement: Supplementary file 1 [file ao5c05079_si_001.pdf]

Investigating the design of ssPalmO-derived lipid nanoparticles for mRNA delivery applications using molecular dynamics simulations

Anjana Barange,[†,‡] Meitram Niraj Luwang,[†,‡] and Santosh Kumar Meena[*,¶]

†*Chemical Engineering and Process Development Division, CSIR-National Chemical Laboratory (NCL), Dr. Homi Bhabha Road, Pune-411008, India*

‡*Academy of Scientific and Innovative Research (AcSIR), Ghaziabad- 201002, India*

¶*Department of Chemical Engineering, Indian Institute of Technology (IIT), Ropar 140001, India*

E-mail: skmeena@iitrpr.ac.in

Phone: +91-1881-234007

Intramolecular distances calculations of atomic pairs

The intramolecular distances were calculated using the gmx distance command in GROMACS, where, for example, the distance between N1 and N2 (nitrogen-containing aromatic ring) within the same lipid molecule was measured. Table 3 in the main text presents the average intramolecular distances for various atomic pairs, including N1-N2 (nitrogen-containing aromatic ring), C9-C14 (aromatic ring carbon), O1-O2 and O3-O4 (carbonyl oxygen), C23-C24 and C29-C34 (aromatic ring carbon), O5-O6 and O7-O8, C45-C61 (unsaturated carbon),

and C54-C70 (terminal carbon) for trans-ssPalmO, cis-ssPalmO-phe, trans-ssPalmO-phe, cis-ssPalmO-ben, trans-ssPalmO-ben, and the mixed lipid bilayer. Some of these intramolecular distances are also illustrated in Figure S1.

Intermolecular distances calculations of atomic pairs

The intermolecular distance was determined by calculating the average distance between specific atomic pairs, such as N1-N1 or N2-N2 (the distance between nitrogen-containing aromatic rings from different lipid molecules within the bilayer). This was achieved by measuring the first peak value in the radial distribution function (RDF) of N1-N1. Similarly, the intermolecular distances of other atomic pairs were calculated using the same approach. The intermolecular distances of these atomic pairs are provided in Table 4 of the main text. The RDF as a function of distance for N1-N1 or N2-N2, unsaturated carbon, and terminal carbon is shown in Figure S2 for all the lipid bilayers."

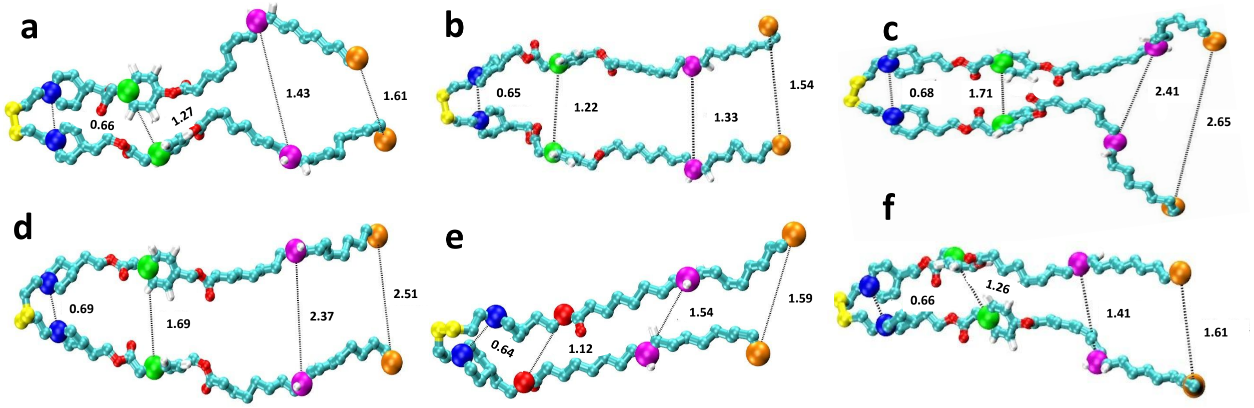

Figure S1: Representation for the intramolecular distance in nm (a) Cis-ssPalmO-phe, (b) Trans-ssPalmO-phe, (c) Cis-ssPalmO-ben, (d) Trans-ssPalmO-ben, (e) Trans-ssPalmO, (f) Cis-ssPalmO-phe from DOPC+Cis-ssPalmO-phe bilayer

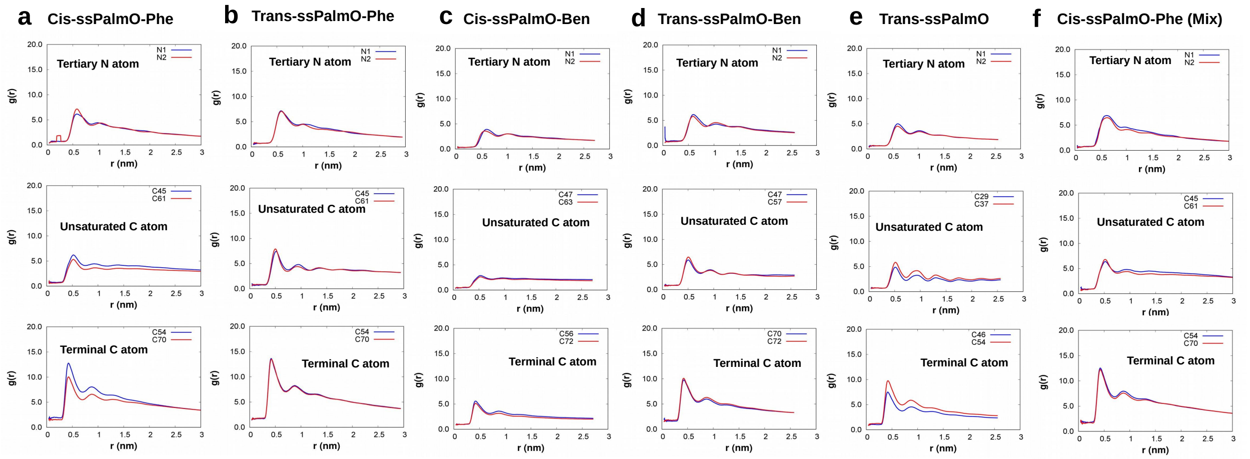

Figure S2: The radial distribution function (RDF) as a function of distance for atomic pair N1-N1 or N2-N2, unsaturated carbon, and terminal carbon are reported for bilayer of (a) Cis-ssPalmO-phe, (b) Trans-ssPalmO-phe, (c) Cis-ssPalmO-ben, (d) Trans-ssPalmO-ben, (e) Trans-ssPalmO, (f) DOPC+Cis-ssPalmO-phe bilayer

Simulation Details of single lipid

For a single lipid, MD simulation was carried out using the GROMACS package (version 2018.1). After adding water, energy was minimized until the highest force applied to any one atom was less than 1000 kJ mol^{-1} nm^{-1}. Within a cut-off length of 1 nm, the Lennard-Jones potentials for van der Waals interactions were computed. The electrostatic interactions were calculated using a summation algorithm; the particle-mesh Ewald (PME) method up to a cutoff distance of 1.0 nm was employed for real-space summation. Using the Parrinello–Rahman barostat, a semi-isotropic pressure coupling system was used to provide a pressure of 1 atm with compressibility of 4.5×10^{-5} bar^{-1}, as implemented in GROMACS during the simulations. A constant temperature of 303.15 K was maintained using the Berendsen thermostat. Under periodic boundary conditions, the simulations were run in rectangular boxes using a Verlet cutoff scheme algorithm for integrating the Newtonian equations of motion, with a time step of 2 fs, and trajectories were saved every 50 ps. We equilibrated the systems in four steps of NVT equilibration (constant particle number, volume, and temperature), each for 1 ns. Then, the equilibrated NVT structure was subjected to NPT equilibration (constant particle number, pressure, and temperature), followed by four steps

of equilibration, each lasting 1 ns. Final production run simulations were performed for 250 ns.

For a single lipid, MD simulation was carried out in a cubic box of dimension 6 x 6 x 6 nm using the same protocol as mentioned above.

Comparison of 1–2 bond pair distances of Cis-ssPalmO-ben obtained from our MD simulations with those derived from dissipative particle dynamics (DPD)

Tanaka *et al.* studied the FMO-DPD simulation for the cis-ssPalmO-ben and calculated the 1–2 bond pair (r_e) in DPD length unit (R_c).[1] The study investigated the mechanism behind aromatic ring-driven membrane destabilization in lipid molecules in an LNP using molecular dynamics simulation and dissipative particle dynamics (DPD) simulation, as well as the fragment molecular orbital (FMO) method for parameter calculation. To determine the time evolution of a cubic cell system, a molecular dynamics simulation was performed using the modified Verlet algorithm. A total of 99,990 beads with a mean density of 3.3 and a cut-off radius of 1 were used in the simulation. The potential energy surface was depicted using the dihedral angle across the S–S bond, with an ideal angle of 85°, and diethyl sulphide was used as the model molecule. 17 segments represent the ssPalmO-ben molecule, and the angles of the scaffolds were defined as C^6-F^0-C^{14}.

To parameterize the DPD model, representative groupings of atoms from GROMOS-based united-atom simulations were mapped to DPD beads. Center of mass distances between these groupings were used to estimate effective bond lengths, which define the DPD equilibrium bond distance r_e. This approach, consistent with prior coarse graining studies, was further supported by radial distribution function (RDF) analysis, which identified the most probable inter-bead distances. Full mapping details are provided in the figure S3. The

ssPalmO-ben structure was divided into 17 segments for simulation purposes. The radial distribution function (RDF) between the relevant grouped atom pairs was used to determine the most probable distance, which was then taken as the effective equilibrium bond length in the DPD model.

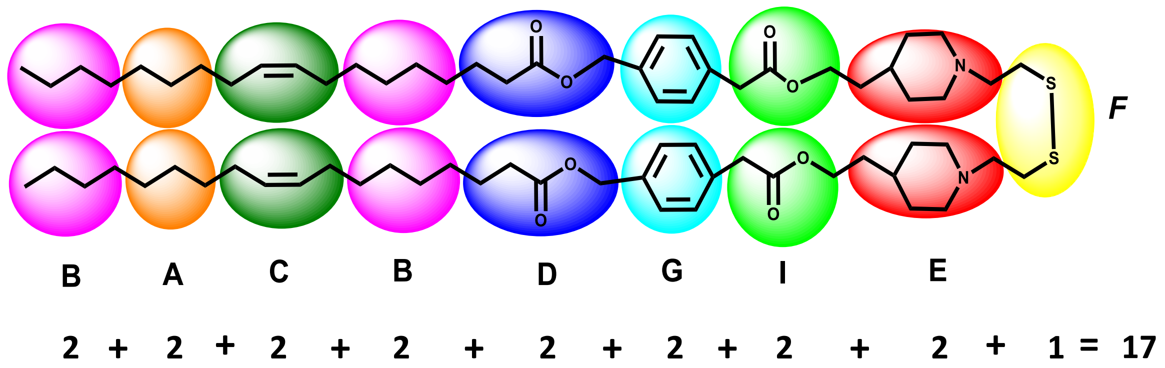

Figure S3: Molecular structure and segmentation of the ssPalmsO-ben

In this paper, we calculated the equilibrium length using the Radial Distribution Function (RDF). The first peak position in the RDF corresponds to the preferred bonding distance, denoted as r_e. Here, we converted the MD-derived r_e to DPD units. In molecular dynamics (MD) simulations, distances are typically expressed in nanometers (nm) or Ångströms (Å), while in dissipative particle dynamics (DPD) simulations, lengths are expressed in units of R_c (cutoff radius). To convert to FMO-DPD, we need to express r_e in DPD units (R_c). For this, we must determine the value of R_c. In this study, we considered the united atom model and selected $R = 0.65$ nm. The formula for converting r_e to DPD units (R_c) is given below The conversion from MD-derived equilibrium distance to DPD units is given by the following equation

$$R_e^{\mathrm{DPD}} = \frac{R_e^{\mathrm{MD}}}{R_c}$$

where R_e^{DPD} is the bond distance in DPD units (R_c units), R_e^{MD} is the bond distance in MD (nm), and R_c is the DPD cutoff radius (nm).

Common values of R_c are:

- $R_c = 0.65$ nm (common for water-like systems)
- $R_c = 1.0$ nm (for larger beads, polymers, etc.)

We observed that our values closely match the DPD simulations, especially considering the different levels of resolution used in both studies (DPD vs atomistic MD)

Table S1: The 1-2 bond pair in DPD unit.

**1-2 bond pair**	**Equilibrium length** r_e (nm)	**MD simulation** r_e (R_c)	**FMO-DPD simulation** r_e (R_c)
F-E	0.51	0.78	0.85
E-I	0.49	0.75	0.81
I-G	0.43	0.66	0.63
G-D	0.49	0.75	0.72
D-B	0.55	0.84	0.80
B-C	0.47	0.72	0.67
C-A	0.40	0.61	0.59

DFT calculation of ssPalmO-phe lipids

We performed DFT calculations of a certain lipid ssPalmO-phe for both its cis and trans configurations to understand its electronic descriptors. Table S2 shows the calculated quantum chemical parameters for this particular lipid in its cis and trans form. The molecular orbital diagrams is shown in Figure S4. The band gap of $\sim$ 6 eV results are in agreement with similar lipids.[2]

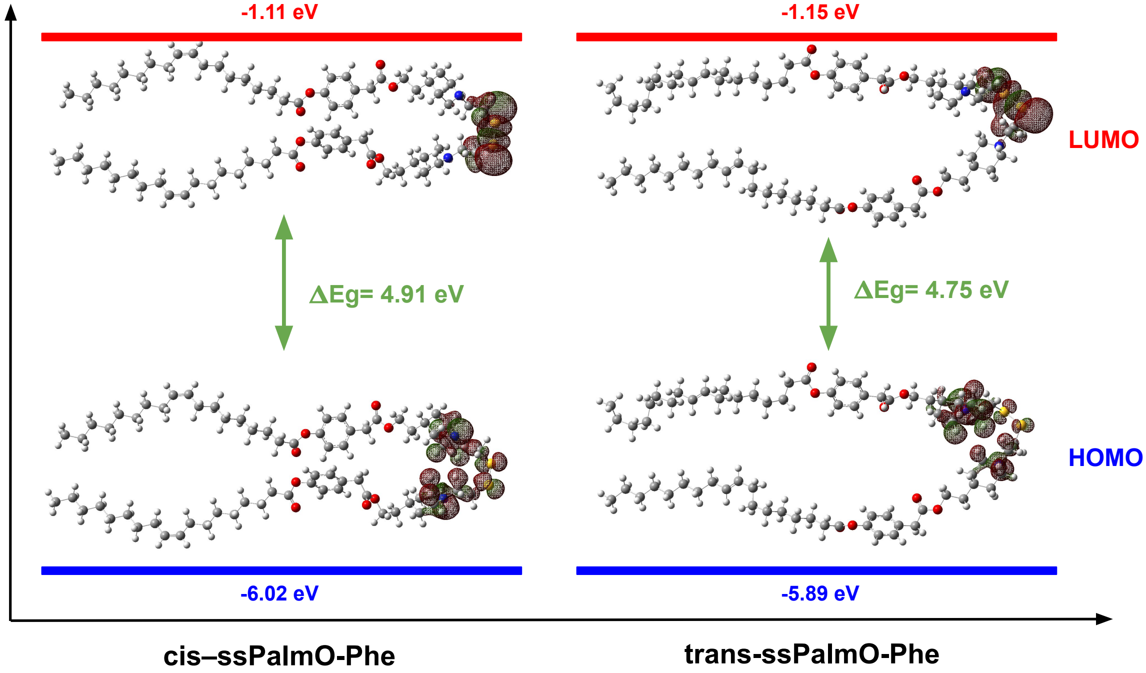

Figure S4: Molecular orbital diagrams of Cis-ssPalmO-phe and Trans-ssPalmO-phe.

Table S2: Computed quantum chemical parameters for Cis-ssPalmO-phe and Trans-sspalmO-phe.

Sl. No.	Molecule	E_{LUMO} (eV)	E_{HOMO} (eV)	ΔE (eV)	η (eV)	χ (eV)	μ (eV)
1.	Cis-ssPalmO-phe	-1.11	-6.02	4.91	2.46	-3.57	3.57
2.	Trans-sspalmO-phe	-1.15	-5.83	4.74	2.37	-3.52	3.52

Chemical structure of lipids

The carbon labels in the figure S5 are mainly used to index atoms in our simulations and may not follow standard IUPAC naming, especially for isomers. The differences in numbering between structures (c)/(d) and (e)/(f) are automatically assigned based on atom connectivity during structure generation and may vary across isomeric forms, even when the underlying chemical composition remains the same. As such, they do not follow standard IUPAC chemical numbering. To avoid confusion and ensure transparency, we have provided the complete structures of all lipids used in this study, including their full atom labeling, in the figure S5. All atoms are now clearly labeled, and chemical bonds are accurately represented to reflect the proper molecular connectivity.

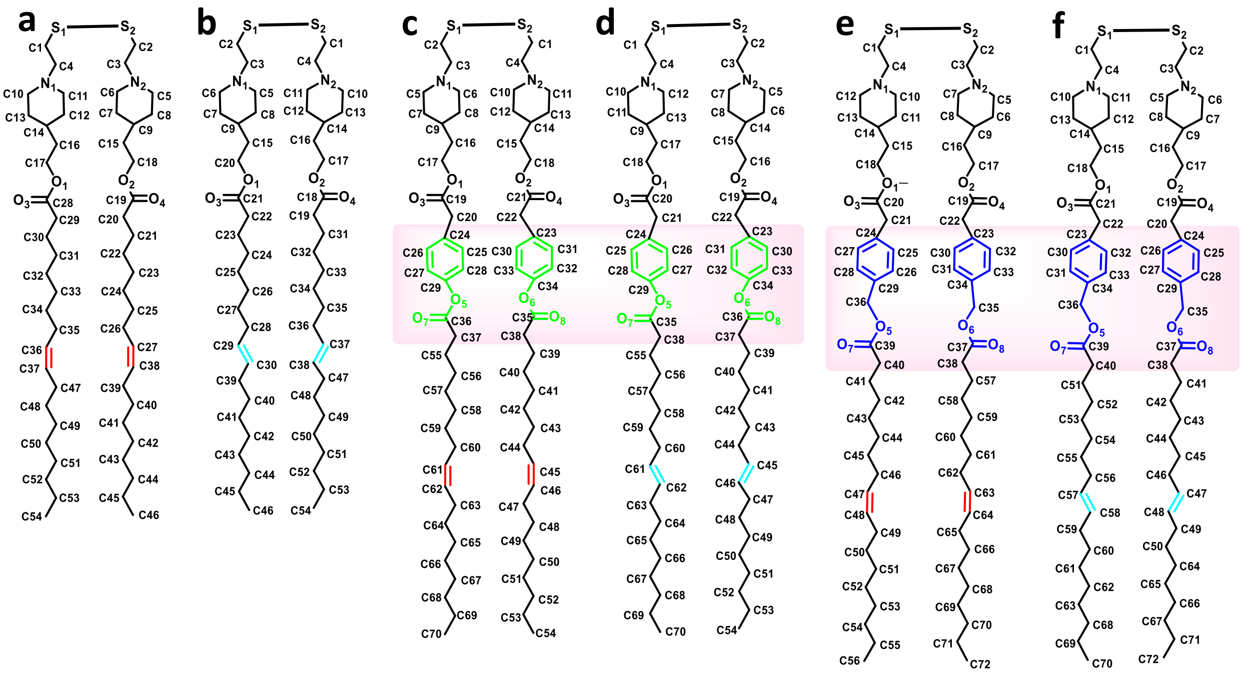

Figure S5: Chemical representation of the lipids and their isomers: (a) Cis-ssPalmO, (b) Trans-ssPalmO, (c) Cis-ssPalmO-phe, (d) Trans-ssPalmO-phe, (e) Cis-ssPalmO-ben, and (f) Trans-ssPalmO-ben.

Table S3: Pharmacokinetic and toxicity properties of the studied lipids.

**Parameter**	**Cis-ssPalmO**	**Trans-ssPalmO**	**Cis-ssPalmO-phe**	**Trans-ssPalmO-phe**	**Cis-ssPalmO-ben**	**Trans-ssPalmO-ben**
Molecular Weight (Da)	1158.72	1165.33	1172.79	1198.84	1200.82	≈1180
logP	14.12	14.38	14.45	14.91	14.91	14.5
TPSA (Å^2)	110.6	108.4	111.7	102.4	111.7	100.4
Caco-2 Permeability (log unit)	-5.19	-5.22	-5.26	-5.27	-5.30	-5.25
MDCK Permeability (log unit)	-4.51	-4.52	-4.70	-4.71	-4.68	-4.72
Plasma Protein Binding (%)	98.42	98.65	98.35	98.34	99.45	≈99
VDss (L/kg)	-0.24	-0.28	-0.23	-0.21	-0.63	-0.27
BBB Penetration	No	No	No	No	No	No
CYP1A2 Inhibition	Yes	Yes	Yes	Yes	Yes	Yes
CYP3A4 Inhibition	Yes	Yes	Partial	No	Yes	Yes
CYP2D6 Inhibition	Yes	Yes	Yes	Yes	Yes	Yes
CYP2B6 Inhibition	Yes	Yes	Yes	Yes	Yes	Yes
HLM Stability	Stable	Stable	Stable	Stable	Stable	Stable
Clearance (mL/min/kg)	3.25	3.31	3.34	3.40	2.96	≈3.2
Half-Life (h)	3.14	3.25	3.10	3.25	3.25	3.20
hERG Inhibition	High	High	High	High	High	High
Skin Sensitization	No	No	No	No	No	No
Carcinogenicity	No	No	No	No	No	No
Genotoxicity	No	No	No	No	No	No
Respiratory Toxicity	0.95	0.91	0.96	0.88	0.95	≈0.93
Nephrotoxicity	0.94	0.97	0.95	1.00	1.00	≈0.97

ADMET prediction

Data collected from ADMETlab 3.0 analyses. The table contains ssPalmO-derived lipid nanoparticles. Values indicate projected pharmacokinetic and toxicological parameters for

lipid nanoparticle formulation design. For all the six ssPalmO derived analogs, in silico ADMET predictions (ADMETlab 3.0) consistently display high molecular weights ($\sim$1100-1200 Da) and logP values ($>$14), suggesting low intrinsic solubility and excessive lipophilicity. All lipids function as stable structural excipients rather than active agents, as evidenced by their minimal blood-brain barrier penetration, strong plasma protein binding ($>$98%), and poor intestinal permeability. The half-life (3 hours) and moderate clearance (3 mL/min/kg) of CYP1A2, CYP2D6, and CYP3A4 inhibition are predicted by metabolic modeling. Although there are no known mutagenic or carcinogenic hazards, toxicity predictions suggest possible hERG inhibition and skin sensitivity. All things considered, the ADMET profiles show good pharmacokinetic compatibility for use as non-reactive, biocompatible carriers in lipid nanoparticle systems.

References

(1) Tanaka, H.; Takahashi, T.; Konishi, M.; Takata, N.; Gomi, M.; Shirane, D.; Miyama, R.; Hagiwara, S.; Yamasaki, Y.; Sakurai, Y.; others Self-degradable lipid-like materials based on "hydrolysis accelerated by the intra-particle enrichment of reactant (HyPER)" for messenger RNA delivery. *Advanced Functional Materials* **2020**, *30*, 1910575.

(2) Peng, H.; Hou, H.-Y.; Chen, X.-B. DFT calculation and Raman spectroscopy studies of α-linolenic acid. *Química Nova* **2021**, *44*, 929–935.
